# A Case of Pediatric Yersinia pseudotuberculosis Infection Complicated by Acute Kidney Injury Successfully Treated With Prednisolone Without Renal Replacement Therapy

**DOI:** 10.7759/cureus.95592

**Published:** 2025-10-28

**Authors:** Takuma Ohnishi, Satoshi Sato, Yoji Uejima, Ippei Miyata, Eisuke Suganuma

**Affiliations:** 1 Department of Infectious Diseases, Immunology, and Allergy, Saitama Children’s Medical Center, Saitama, JPN; 2 Department of Pediatrics, Keio University School of Medicine, Shinjuku, JPN; 3 Department of Pediatrics, Kawasaki Medical School, Kurashiki, JPN

**Keywords:** acute interstitial nephritis, acute kidney injury, prednisolone, renal replacement therapy, yersinia pseudotuberculosis

## Abstract

*Yersinia pseudotuberculosis *(*Y. pstb*)* *infection causes bacterial gastroenteritis and may lead to complications, including acute kidney injury (AKI). We report a case of a previously healthy three-year-old boy presenting with prolonged fever, abdominal distention, generalized edema, and impaired renal function following gastroenteritis symptoms. Laboratory tests indicated severe inflammation and AKI, classified as renal failure by the pediatric-modified Risk, Injury, Failure, Loss, End-Stage Renal Disease (RIFLE) AKI classification system. Intravenous prednisolone rapidly improved clinical symptoms and renal function, eliminating the need for renal replacement therapy. Elevated serum anti-*Y. pstb*-derived mitogen (YPM) antibody titers confirmed the diagnosis. Pediatric AKI due to *Y. pstb* infection may involve immune-mediated mechanisms triggered by YPM. In this case, systemic glucocorticoid therapy effectively controlled the inflammatory response and restored renal function, without dialysis. Together with prior evidence that the YPM drives superantigenic immune activation in severe presentations, this case supports consideration of prednisolone as a dialysis-sparing option in select pediatric *Y. pstb*-associated AKI.

## Introduction

*Yersinia pseudotuberculosis *(*Y. pstb*) is a zoonotic enteropathogen acquired primarily via the fecal-oral route from contaminated food, water, or animal contact [1]. Although many infections are self-limited gastroenteritis, *Y. pstb* can trigger systemic hyperinflammatory syndromes, present as gastroenteritis, and can be associated with multiple complications, including terminal ileitis, erythema nodosum, arthritis, septicemia, and Kawasaki disease [1]. A central driver is *Y. pstb*-derived mitogen (YPM), a superantigen that causes T‑cell activation and cytokine release. Human studies show higher anti-YPM titers and characteristic T-cell signatures in patients with systemic manifestations, including transient renal dysfunction, compared with those with localized disease [2].

Renal involvement typically reflects acute interstitial nephritis (AIN) leading to acute kidney injury (AKI), often managed supportively; however, dialysis is occasionally required. Pediatric cases of *Y. pstb*-associated AKI have been reported in Japan and Korea [3-5]. Previous literature from Korea reported that AKI developed in 13.6% of patients with *Y. pstb* infections; among these, 2-14% required renal replacement therapy [6]. In this report, we present a pediatric case of AKI secondary to *Y. pstb* infection successfully managed with glucocorticoid therapy. We believe this is the first reported case of *Y. pstb*-associated AKI treated with systemic prednisolone.

## Case presentation

A three-year-old boy with remittent fever, abdominal pain, and generalized edema was transferred to our center (patient presentation: November 2020). He was initially seen at a community hospital with complaints of remittent fever associated with abdominal pain, without vomiting or diarrhea. Abdominal ultrasonography at that time revealed an edematous intestinal wall, and he was diagnosed with gastroenteritis. He was started on cefotaxime on day five of his illness. The patient had no history of eating raw meat, drinking well water, playing in a lake or pond, having contact with animals, or recent travel abroad. Despite three days of cefotaxime therapy, his symptoms progressed, and he developed renal dysfunction. On day eight of illness, he was transferred to our medical center for further evaluation. On admission, he was alert and oriented but lethargic. His vital signs were as follows: body temperature, 40.5°C; heart rate, 140 bpm; and blood pressure, 108/59 mmHg. Physical examination revealed a distended, tender abdomen and generalized edema, most prominent in the face and legs. No skin rashes were noted.

Blood examination revealed leukocytosis (25,940/µL; neutrophils, 88.9%; lymphocytes, 8.0%; eosinophils, 0.5%), hypoproteinemia (total protein, 4.5 g/dL; albumin, 1.6 g/dL), and elevated C-reactive protein (CRP) (21.31 mg/dL) and ferritin (458.7 ng/mL) levels. Although his urine output rate remained over 1 mL/kg/h, AKI was suspected based on the blood chemistry examination results. He had a creatinine (Cr) of 1.06 mg/dL, Na^+^ of 130 mEq/L, K^+^ of 3.8 mEq/L, and phosphate of 2.8 mg/dL. Urinalysis revealed proteinuria, hematuria, and glucosuria but no eosinophiluria. Microscopic examination of the urine demonstrated pyuria (30-49 cells/high power field) without bacteriuria. He had an estimated glomerular filtration rate (eGFR) of 31.36 mL/min/1.73 m^2^, fractional excretion of Na of 1.74%, and urinary β_2_-microglobulin of 75,476 µg/L (Table 1).

**Table 1:** Laboratory findings on admission to our hospital (day eight of illness).

Parameters	Patient values	Reference value
Complete Blood Count		
White blood cells (x10^3^/µL)	25.9	11.5–15.5
Neutrophil (%)	88.9	23–45
Lymph (%)	8.0	35–65
Eosinophils (%)	0.5	0–3
Blood Chemistry		
Protein, total (g/dL)	4.5	5.9–7.0
Albumin (g/dL)	1.6	3.4–5.2
C-reactive protein (mg/dL)	21.31	< 0.8
Ferritin (ng/mL)	458.7	10.0-99.9
Creatinine (mg/dL)	1.06	0.1–0.4
Sodium (mEq/L)	130	135–145
Potassium (mEq/L)	3.8	3.5–5.2
Phosphorus (mg/dL)	2.8	3.8–6.5
Urinalysis		
Protein/Creatinine ratio	1.85	< 0.2
White blood cells (/high power field)	30–49	0–5
Kidney function		
Estimated glomerular filtration rate (mL/min/1.73 m^2^)	31.36	> 90
β_2_-microglobulin (µg/L)	75,476	< 290

**Figure 1:**
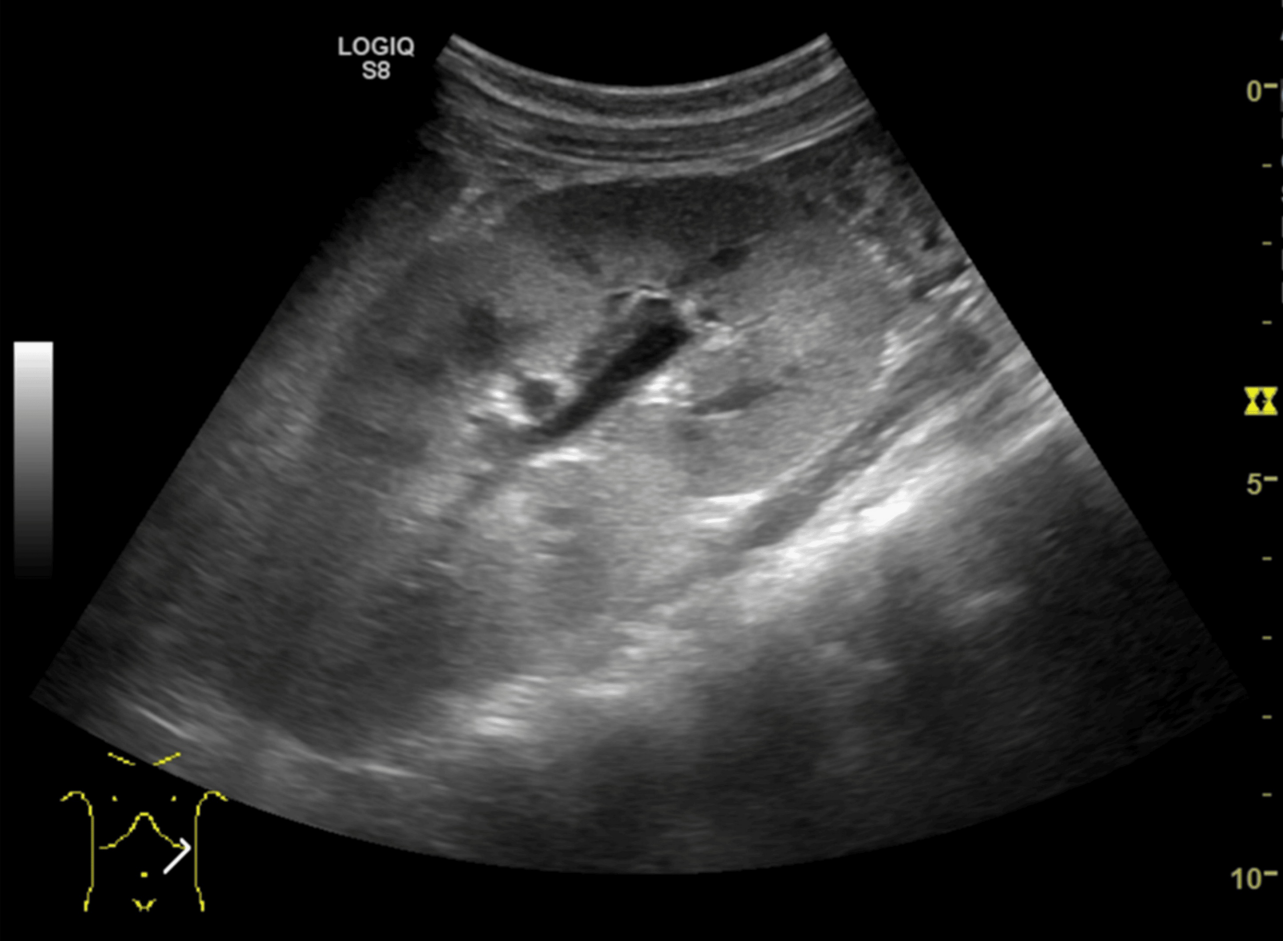
Ultrasound image of the left kidney showing increased echogenicity of the renal parenchyma and renal swelling.

The patient had renal failure based on the AKI classification of the pediatric-modified Risk, Injury, Failure, Loss, End-Stage Renal Disease (RIFLE) criteria [7]. Pediatric-RIFLE failure is defined as ≥75% decline in estimated creatinine clearance (eCCl) or eCCl <35 mL/min/1.73 m², or urine output <0.3 mL/kg/h for 24 h or anuria for 12 h. Our patient met the failure criterion by eGFR alone while maintaining urine output >1 mL/kg/h. The blood and urine culture results were negative; a stool culture was not obtained. Ultrasonographic examination of the kidneys revealed increased renal parenchymal echogenicity and renal swelling (Figure 1).

**Figure 2:**
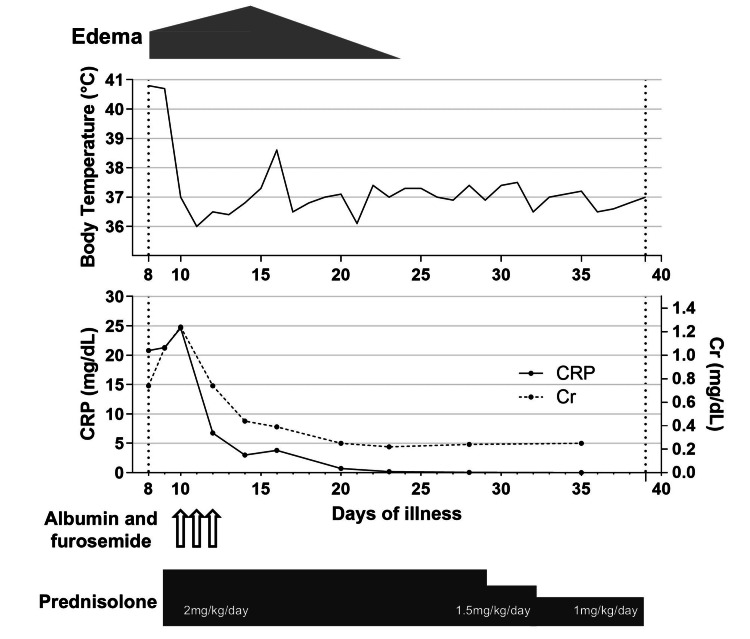
Clinical course of the patient after admission. The patient was transferred to our hospital on day eight of illness and discharged on day 39. The upper panel shows body temperature trends, and the lower panel displays serum levels of C-reactive protein (CRP; solid line, left y-axis) and creatinine (Cr; dashed line, right y-axis). The gray gradient bar at the top represents the severity of edema. Intravenous albumin and furosemide were administered on days nine, 10, and 11. Prednisolone was initiated at 2 mg/kg/day and tapered to 1.5 mg/kg/day and 1 mg/kg/day over time. CRP: C-reactive protein; Cr: creatinine.

The clinical course of the patient is illustrated in Figure 2. Antimicrobial therapy was discontinued on admission to our center. Since the edema and renal function gradually worsened (Cr, 1.24 mg/dL), intravenous prednisolone (2 mg/kg/day) was initiated on day nine of illness. After receiving prednisolone, the patient became afebrile. Albumin (5 g/day) and furosemide (0.5 mg/kg/day) were administered to manage the hypoalbuminemia and edema for three days. The patient’s renal function improved first, followed by the onset of a diuretic phase, after which the generalized edema gradually resolved. Oral prednisolone therapy was initiated on day 13 of illness. However, on day 15, the patient developed a transient fever. Prednisolone administration was switched back to the intravenous route, and intravenous ampicillin/sulbactam (200 mg/kg/day) was administered until day 21. The patient remained afebrile, and intravenous prednisolone was switched back to oral prednisolone and gradually tapered without relapse. The patient was discharged on day 39 of illness.

Serum anti-YPM antibody titers were evaluated by enzyme-linked immunosorbent assay [8]. A 160-fold increase was observed between serum collected on the first and third weeks of the disease, confirming a *Y. pstb* infection.

The day after discharge, oral prednisolone was reduced to 0.5 mg/kg/day and discontinued two weeks later. He was followed in the outpatient clinic for six months after discharge, with no recurrence or sequelae during follow-up.

## Discussion

This is a previously healthy three-year-old with *Y. pstb*-associated AKI whose clinical and laboratory features suggested hypercytokinemia and tubular injury. He improved with systemic prednisolone and did not require dialysis.

The constellation of high fever, edema, markedly elevated CRP and ferritin, sterile pyuria, and tubular injury markers points to hypercytokinemia. Mechanistically, YPM acts as a superantigen, causing broad T-cell activation and cytokine release. In a prospective human study, anti-YPM titers and Vβ3 T-cell expansion were significantly higher in patients with systemic disease than in those with localized gastroenteritis, and these abnormalities diminished in convalescence [2,9,10]. The polyclonal activation of T-cells caused by YPM results in the overproduction of proinflammatory cytokines, which contribute to systemic damage, as seen in Kawasaki disease, reactive arthritis, and interstitial nephritis [9-11].

Pediatric cases of *Y. pstb*-associated AKI have been reported in Japan and Korea [3-5]. Although *Y. pstb*-associated AKI is generally self-limiting and managed with supportive care [4], it can occasionally progress to a severity that necessitates renal replacement therapy, such as hemodialysis [6,11]. In the present case, hypercytokinemia was suspected based on the laboratory data on admission. Prednisolone was administered to regulate the inflammatory cytokines, and its effectiveness was seen clinically and in the decreased CRP and ferritin levels. This case demonstrates that renal replacement therapy can be avoided by administering systemic prednisolone, even in a patient with renal failure based on the pediatric-modified RIFLE criteria [7].

Previous literature reported that fever was the first presenting symptom in patients with AKI associated with a *Y. pstb* infection, followed by gastrointestinal symptoms and AKI approximately six days later [4]. Our patient followed a similar clinical course, with abdominal symptoms following fever, and finally with AKI developing approximately one week from the onset.

In children with AKI associated with a *Y. pstb* infection, most renal histopathological examinations exhibit findings of AIN [12]. Although a renal biopsy was not performed in our case, abnormal urine findings, sterile pyuria, and signs of decreased tubular reabsorptive capacity suggested AIN [13]. The ultrasonographic examination also revealed characteristic features of AIN, including increased renal parenchymal echogenicity and renal swelling, consistent with previous reports [14,15]. AIN is frequently associated with medications, and cephalosporins are among the significant causative agents [13]. However, drug-induced AKI was unlikely in this case due to the following reasons: First, classic drug eruption is suggestive of drug-induced AIN, whereas our patient presented with no skin rashes. Second, an elevated serum eosinophil count, diagnostic for AIN caused by allergic or hypersensitivity reactions to medications [13], was not observed.

Although most reported cases of *Y. pstb*-associated AKI have been managed conservatively with supportive care, our case highlights that systemic glucocorticoid therapy could be a viable treatment option to avoid renal replacement therapy in AKI secondary to *Y. pstb* infection.

## Conclusions

This case highlights the potentially steroid-responsive nature of AKI associated with *Y. pstb* infection. To our knowledge, this is the first reported pediatric case of *Y. pstb*-associated AKI that responded favorably to systemic glucocorticoid therapy. Early recognition of immune-mediated kidney involvement and prompt initiation of anti-inflammatory treatment may prevent the need for renal replacement therapy. Clinicians should consider glucocorticoid therapy as a viable option in similar cases of severe pediatric AKI with suspected inflammatory mechanisms.
